# A biomechanical analysis of different meshes for reconstructions of the pelvic floor in the porcine model

**DOI:** 10.1007/s00404-021-06344-9

**Published:** 2021-11-29

**Authors:** Nadja Trageser, Axel Sauerwald, Sebastian Ludwig, Wolfram Malter, Kilian Wegmann, Leonidas Karapanos, Julia Radosa, Alina Katharina Jansen, Christian Eichler

**Affiliations:** 1 Department of Gynecology and Obstetrics, Faculty of Medicine and University Hospital Cologne, University of Cologne, Kerpener Straße 62, 50931 Cologne, Germany; 2 Department of Gynecology and Obstetrics, St. Marien Hospital Düren, Duren, Germany; 3 Department for Trauma, Hand and Elbow Surgery, Faculty of Medicine and University Hospital Cologne, University of Cologne, Cologne, Germany; 4 Department of Urology, Uro-Oncology, Robot-Assisted and Reconstructive Surgery, Faculty of Medicine and University Hospital Cologne, University of Cologne, Cologne, Germany; 5 Department for Gynecology, Obstetrics and Reproductive Medicine, Saarland University Hospital, Homburg, Germany; 6 Breast Cancer Center, St. Franziskus-Hospital Münster, Hohenzollernring 70, 48145 Münster, Germany

**Keywords:** Biomechanical testing, Laparoscopy, Pelvic organ prolapse, Polypropylene mesh, Sacrocervicopexy, Uro-gynecological surgery

## Abstract

**Purpose:**

Many different surgical approaches have been established for the repair of a pelvic organ prolapse. Especially in laparoscopic surgery, it is important to generate easy surgical techniques with similar stability. This study shall simplify the choice of mesh by evaluating three polypropylene meshes regarding their biomechanical properties.

**Methods:**

Biomechanical testing was performed in the porcine model. The meshes are fixated on porcine fresh cadaver cervices after subtotal hysterectomy. The apical part of the mesh is fixated with parallel screw clamps at the testing frame. Forty-one trials were performed overall, subdivided into four subgroups. The groups differ in mesh type and fixation method. Maximum load, displacement at failure and stiffness parameters were evaluated with an Instron 5565^®^ test frame.

**Results:**

SERATEX^®^ E11 PA (E11) showed the highest values for maximum load (199 ± 29N), failure displacement (71 ± 12 mm) and stiffness (3.93 ± 0.59 N/mm). There was no significant difference in all three evaluated parameters between SERATEX^®^ B3 PA (B3) and SERATEX^®^ SlimSling^®^ with bilateral fixation (SSB). SERATEX^®^ SlimSling^®^ with unilateral fixation (SSU) had the lowest stiffness (0.91 ± 0.19 N/mm) and maximum load (30 ± 2 N) but no significant difference in displacement at failure.

**Conclusion:**

All meshes achieved a good tensile strength, but the results of maximum load show that the E11 is superior to the other meshes. Through a bilateral fixation of SERATEX^®^ SlimSling^®^, a simple operating method is generated without a loss of stability.

## Introduction

Pelvic organ prolapse is a frequent problem which occurs in nearly 50% of parous women [1]. According to DeLancey, the most frequent causal circumstances are failures of connective tissue and muscles of the pelvic floor [2], what can result in sensation of pressure on the vagina, sexual dysfunction or the impairment of the normal function of the urinary tract. In part, these symptoms can be alleviated by conservative methods like pelvic muscle exercises or a vaginal pessary. If the symptoms do not improve adequately with nonsurgical therapy, surgical therapies may be considered [3]. Of all women, 11–19% require surgery during their lifetime [4]. Various surgical techniques are available for the correction of pelvic organ prolapse. The choice of procedure is made based on the patients’ individual characteristics of the disease and the medical history. These include the type and severity of the patient’s symptoms, as well as the patient’s health condition and the severity of the prolapse [3]. The surgical treatment methods may be accompanied by a (sub-) total hysterectomy, but if indicated, uterus-preserving surgery may also be an option [3, 5]. Sacral apical fixation, regardless of being approached through abdominal or laparoscopic methods, is an effective procedure for the correction of symptomatic pelvic organ prolapse [6, 7]. As an example of sacrocolpopexy, success rates of more than 90% for the abdominal group and approximately 80% for the laparoscopic group are reported [6]. The benefits of the laparoscopic method are reduced blood loss during surgery as well as lower morbidity, but it is also accompanied by a longer operating time and additional costs [8, 9].

Even if abdominal sacrocolpopexy is the most common technique, laparoscopic and robotic-assisted methods have become more popular over the years. Besides this, additional approaches for the repair of pelvic organ prolapses were established, like pectopexy, sacrocervicopexy, sacrohysteropexy or sacral colpoperineopexy [10–13]. In this study, a sacrocervicopexy with a supracervical hysterectomy is simulated in an in vitro setting. Sacrocervicopexy achieves high success rates similar to laparoscopic sacrocolpopexy and the subtotal hysterectomy reduces the risk for mesh erosion compared to a total hysterectomy [13].

In the current literature, there is no consensus on a consistent surgical approach for the correction of a pelvic organ prolapse [14, 15]. However, the rough process is similar for several methods. After the preparation of the promontory, the vaginal vault and the parasigmoidal and para-rectal fossa, an implant can be fixated to the vaginal vault or the cervix and the promontory. When fixation is completed, most surgeons perform a reperitonealization [9, 15]. Almost all steps are not standardized [7], starting with the type of mesh. Furthermore, the suture materials and the surgical techniques are variable [15, 16].

The preferences in the selection of a surgical thread vary widely between permanent or delayed [5], as well as absorbable or non-absorbable materials [16, 17]. Likewise, several interposition grafts are available for the fixation of the uterus or cervix, such as allograft, autologous or synthetic materials [4, 18]. Most of the surgeons use a polypropylene mesh [4, 5, 7], which reduces the feasibility of a prolapse recurrence [5] and leads to a higher anatomic durability [19]. The meshes also differ regarding their shape. Dual-pieced meshes are frequently used. The two mesh arms can be fixated at the posterior and anterior part of the vaginal wall. Ordinary single-piece meshes or a single piece with a Y-configuration can be used alternatively [7].

In this study, two dual-piece, beaked meshes (SERATEX^®^ E11 PA (E11) and SERATEX^®^ B3 PA (B3)) and a narrow single-piece mesh (SERATEX^®^ SlimSling^®^) were compared with each other regarding their biomechanical properties. Therefore, the parameters stiffness, maximum applicable load, and displacement at failure, as well as the limiting factors of the fixation methods were determined. SERATEX^®^ SlimSling^®^ is a combination of suture material and textile implant. It reduces the number of sutures and separate suture material is no longer required. Since laparoscopic suturing is a time-consuming task [20], this mesh could reduce the operation time.

Biomechanical comparisons between SERATEX^®^ SlimSling^®^, E11 and B3 have not been described so far, this is the first comparative clinical data.

Given the difficulties urogynecologist have, to opt for a mesh in pelvic organ prolapse repair, this study should simplify the choice of mesh in the clinical setting. According to the manufacturer, all meshes can be used equally for the correction of pelvic organ prolapse.

## Methods

Forty-one fresh, unfrozen, porcine cadaver uteri were used for the fixation method evaluation. The mesh fixation on the cervix was performed after a supracervical hysterectomy and the preparation of vagina and cervix. Every preparation was used only once which resulted in a need of 41 meshes in total.

**Fig. 1:**
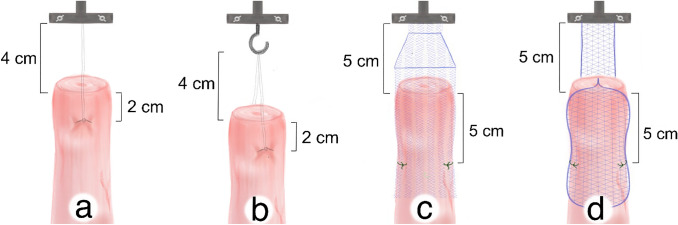
Testing setup. **a** SERATEX^®^ SlimSling^®^ with unilateral fixation. **b** The same mesh with a bilateral fixation. **c** SERATEX^®^ E11 PA and **d** SERATEX^®^ B3 PA

**Fig. 2:**
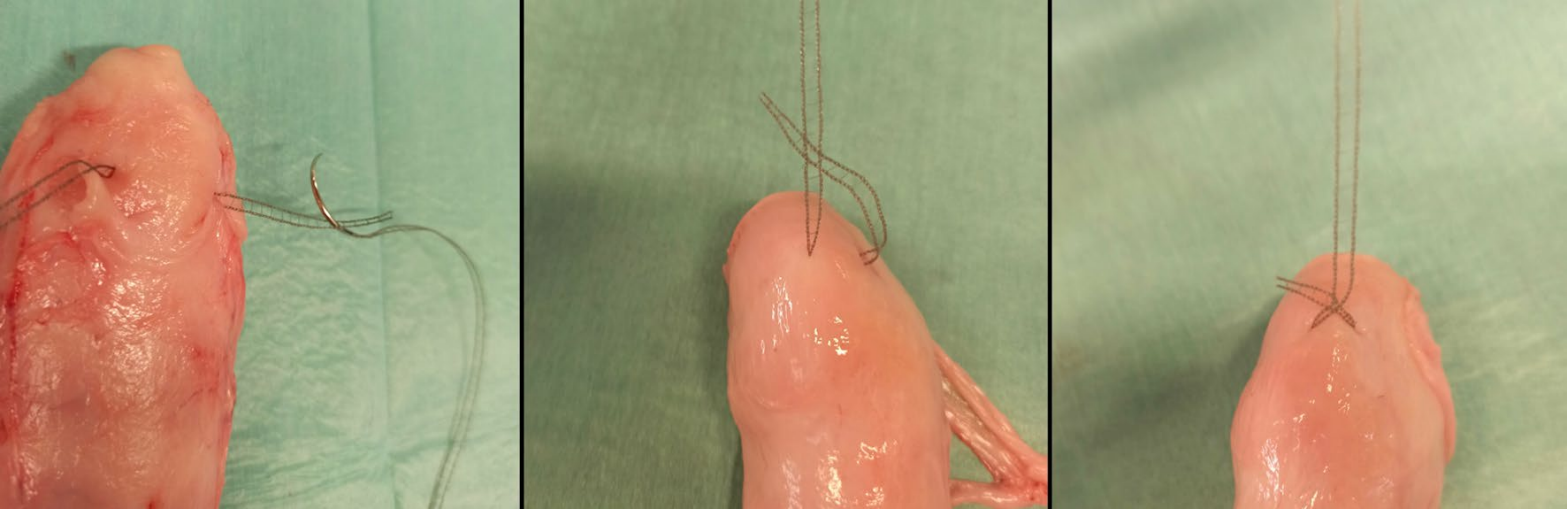
Knotting method for SERATEX^®^ SlimSling^®^. Instead of a common surgical knot, the needle is threaded through the fifth loop of the mesh

The 41 trials can each be assigned to one of four groups. Group 1 (*n* = 12) evaluated the SERATEX^®^ SlimSling^®^ (0.3 × 90 cm—SERAG-WIESSNER GmbH & Co. KG, Naila, Germany) with a unilateral fixation (SSU). SERATEX^®^ SlimSling^®^ is utilized as a combination of suture material and textile implant, so that no separate suture is necessary. It is made up of two knitted strands of polypropylene fibers running parallel to the tensile load. The strands are connected via a single fiber with a diameter of 0.14 mm. The mesh has a pore size of 2 × 1.5 mm. Figure 1a demonstrates the SSU group. The mesh was fixed at the cervix, two cm distal of the surgical margin. Instead of a common surgical knot, the needle was threaded through the fifth loop of the mesh, cf. Figure 2. Threading could lead to a reduced operation time, as regular knotting is a time-consuming task in laparoscopic surgery [20].

Group 2 (*n* = 10) contained the same mesh with a bilateral fixation (SSB). The two stitches were positioned opposite each other. Figure 1b shows the bilateral fixation. One side of the mesh was fixated as pictured in Fig. 2. The other side was fixed with a surgical knot, two cm distal of the surgical margin as well.

Group 3 (*n* = 9) assessed E11 (5.5 × 26 cm—SERAG-WIESSNER GmbH & Co. KG, Naila, Germany), a dyed, monofilament, partially absorbable mesh with a pore size of 2 × 3 mm. The mesh is made of knitted monofilament polypropylene fibers, which follow oblique to the tensile load and have a diameter of 0.15 mm. In Fig. 1c, the fixation of E11 is pictured. Group 4 (*n* = 10) used B3 (4.5 × 26 cm—SERAG-WIESSNER GmbH & Co. KG, Naila, Germany), which is also dyed, monofilament and partially absorbable. As opposed to the previous mesh, six single, non-knitted, longitudinally placed polypropylene fibers take up the force in the direction of tensile load. They have a diameter of 0.19 mm. Fibers of different diameters are intertwined with the longitudinal ones. The pore size is 2 × 4 mm. Figure 1d shows the attachment of B3.

**Fig. 3:**
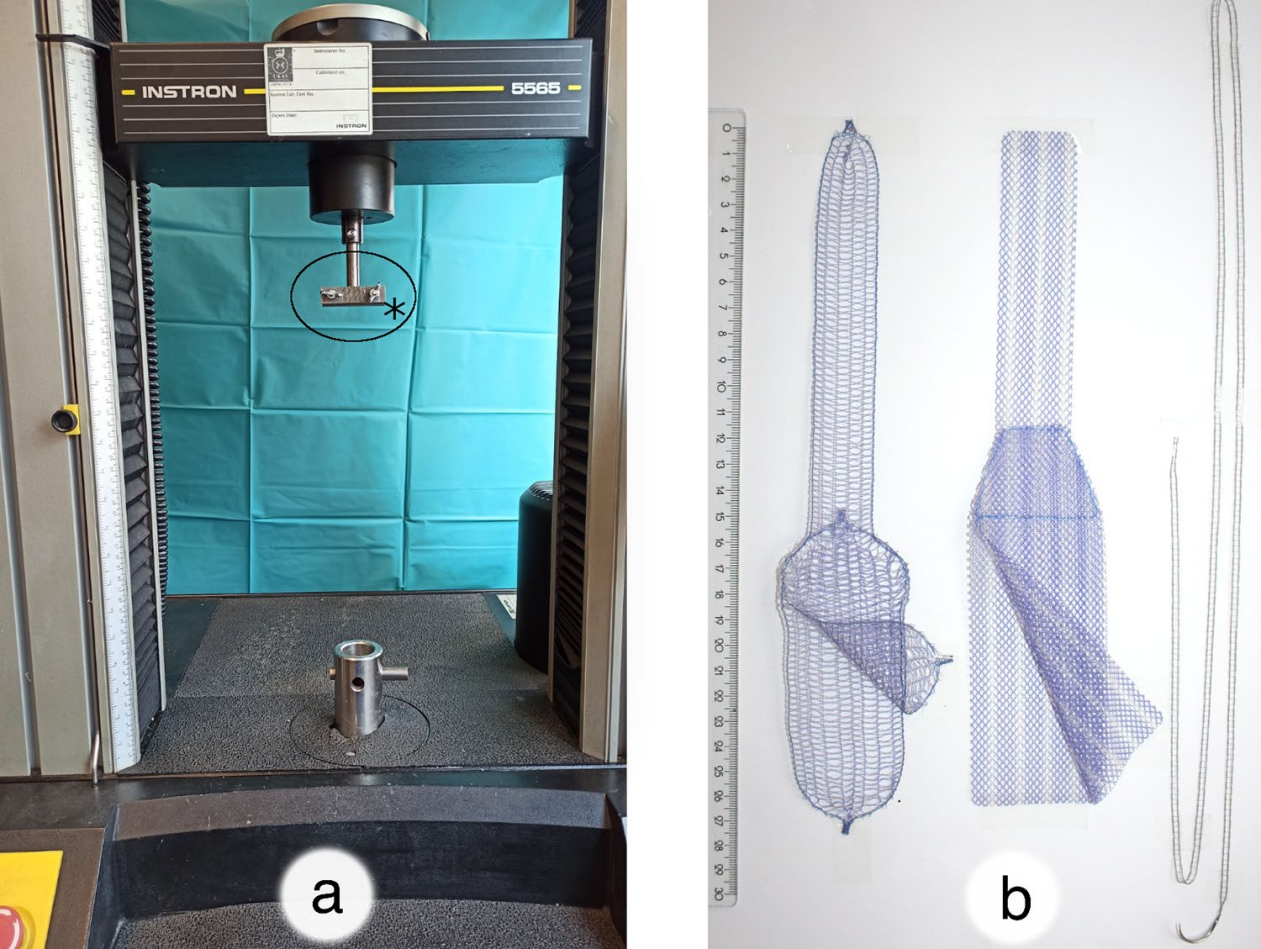
Testing frame and evaluated meshes. **a** Shown is the testing frame that was used. The black asterisk marks the parallel screw clamps that were used for the apical fixation of the meshes. **b** All three evaluated meshes are shown. From left to right: SERATEX^®^ B3 PA, SERATEX^®^ E11 PA, SERATEX^®^ SlimSling^®^ (by courtesy of SERAG-WIESSNER GmbH & Co. KG)

Both meshes, E11 and B3, were fixed with two single interrupted sutures, 5 cm distal of the surgical margin on each side, respectively. Every knot was tied in the same manner, in terms of a surgical knot, with a stitch including four pores of the mesh. The long arm of E11 was trimmed in the same width as the long arm of B3. For groups 3 and 4, a synthetic, braided, non-absorbable PremiCron^®^ suture 1 with a HR26s needle and 75 cm green filament (B. Braun Medical Ltd, Sheffield, United Kingdom) was used. In all four groups, the sacral fixation was simulated by the Instron 5565^®^ testing frame. The meshes were fixed between the porcine cervix and the testing frame by using sutures for cervical fixation and screw clamps (SSU, E11 and B3) or hooks (SSB) for apical fixation. The screw clamps are shown in Fig. 3a and also indicated in Fig. 1. A model of the hook can be seen in Fig. 1. Analysis was accomplished on an Instron 5565^®^ testing frame with the aid of the Bluehill^®^ 2 Software. Figure 3a shows the testing frame that was used and Fig. 3b shows all three meshes (by courtesy of SERAG-WIESSNER GmbH & Co. KG, Naila, Germany).

Initially, 12 trials per group and a total of 48 trials were planned. In the first trials, the meshes were damaged by the screw clamp at the apical fixation. The edge of the screw clamps exerted biomechanical forces on the mesh that did not correlate with those in the female pelvis. On this account, the aforementioned trials have not been suitable to evaluate the research questions of our study and were excluded from the analysis. By padding the clamp, the outlined problem could be solved, and the following trials could be used for the evaluation. However, the initial difficulties resulted in a different number of trials per group (Group 1 *n* = 12, Group 2 *n* = 10, Group 3 *n* = 9, Group 4 *n* = 10).

A load increase of 5 N/s and a preload of 5 N was performed for all transient evaluations of the fixation methods. The Instron 5565^®^ test frame directly measured maximum load and displacement at failure. Based on the machine data in terms of load (N) and displacement (mm), a force–deflection graph was created. By determining the slope in the linear region of the curve, the calculation of stiffness (N/mm) was executed. Stiffness characterizes the rigidity of the mesh and indicates the extent to which the mesh can resist deformation under the pull-out force. This does not always correspond with the physiologically relevant area. The maximum load is the highest force that the mesh could sustain before it failed. It was defined as the highest point of the force–deflection graph, before the first clear gradient decline in the curve. The displacement at failure specifies the elongation of the construct in mm at the time when maximum load is reached. The displacement at failure is the increase in the length of the mesh from the beginning of the trial to the point of failure.

### Statistics

Vassar Stats^®^ (Vassar College, Poughkeepsie, NY, USA) statistics program was used to conduct the statistical analysis. ANOVA analysis was used to evaluate significances when appropriate. Significance was defined as *p* < 0.05.

## Results

**Fig. 4:**
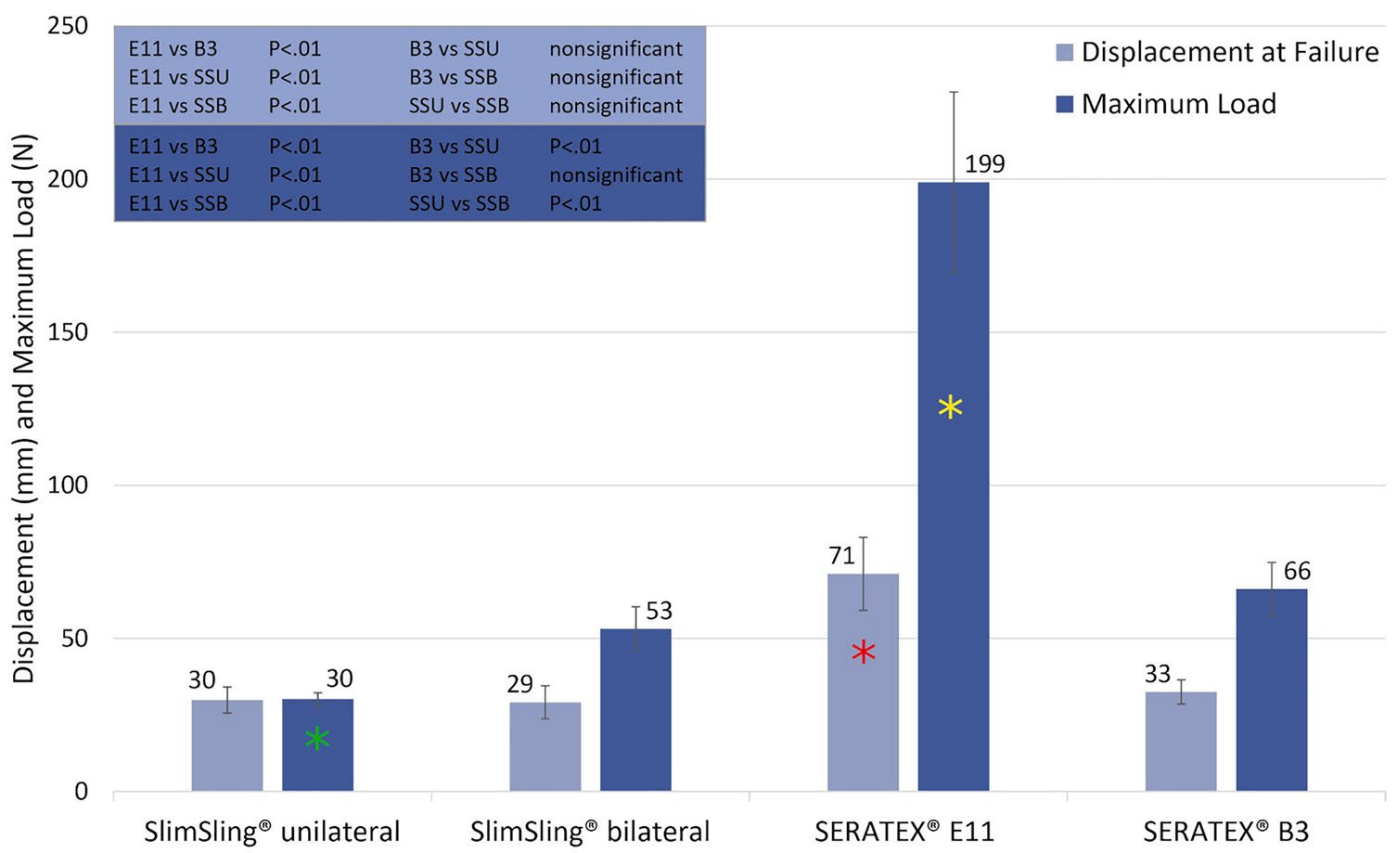
Comparison of all evaluated groups for displacement at failure and maximum load. Shown are the results of displacement at failure and maximum load for all evaluated groups. Error bars represent standard deviations. The blue box in the upper left of the figure shows the results of the ANOVA analysis. *SSU* SERATEX^®^ SlimSling^®^ with unilateral fixation, *SSB*  SERATEX^®^ SlimSling^®^ with bilateral fixation, *E11* SERATEX^®^ E11 PA, *B3* SERATEX^®^ B3 PA. The green asterisk marks the maximum load of SERATEX^®^ SlimSling^®^ with unilateral fixation. It is significantly lower than maximum loads generated by the other fixation methods. The red asterisk marks the displacement at failure of SERATEX^®^ E11 PA. The displacement is significantly higher than displacements generated by the other fixation methods. The yellow asterisk marks the maximum load of SERATEX^®^ E11 PA. It is significantly higher than maximum loads generated by the other fixation methods

**Fig. 5:**
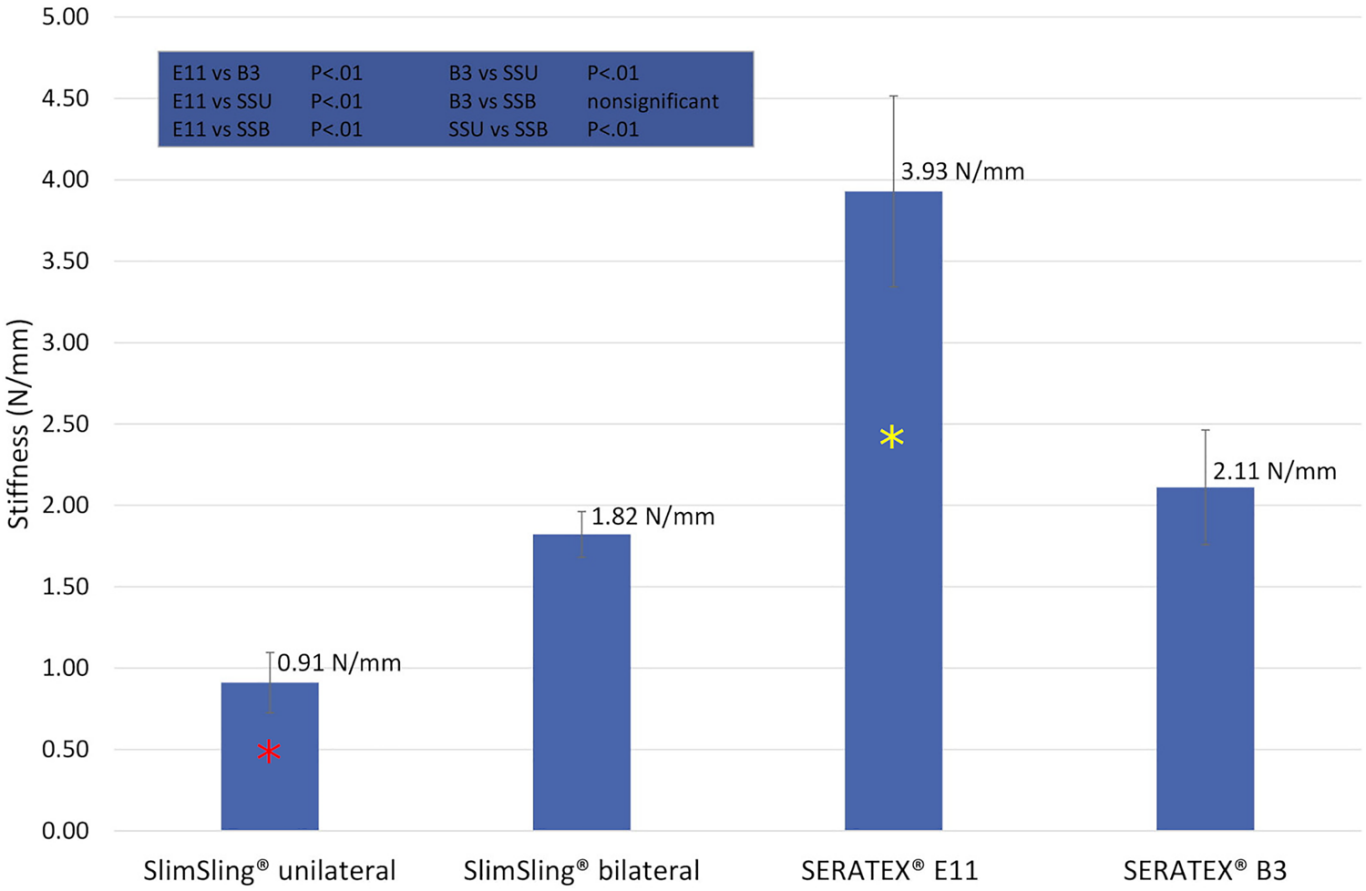
Comparison of all evaluated groups for the parameter stiffness. Error bars represent standard deviations. The blue box in the upper left of the figure shows the results of the ANOVA analysis. *SSU* SERATEX^®^ SlimSling^®^ with unilateral fixation, *SSB* SERATEX^®^ SlimSling^®^ with bilateral fixation, *E11* SERATEX^®^ E11 PA, *B3* SERATEX^®^ B3 PA. The red asterisk marks the evaluated value of the parameter stiffness of SERATEX^®^ SlimSling^®^ with unilateral fixation. It is significantly lower than the stiffness of the other meshes. The yellow asterisk marks the evaluated value of the parameter stiffness of SERATEX^®^ E11 PA. It is significantly higher than the stiffness of the other meshes

Overall, 41 trials subdivided into four subgroups were performed. All partial results are registered in Table 1. *p* values of the intergroup ANOVA analysis are recorded in Figs. 4 and 5.

**Table 1:** Overall results for all four evaluated groups

Evaluated entity Total trials = 41	*n*	Displacement at failure (mm)	Maximum load (N)	Stiffness (N/mm)	Failure mode
SlimSling^®^ unilateral fixation	12	30 (± 4)	30 (± 2)	0.91 (± 0.19)	Mesh failure (12/12)
SlimSling^®^ bilateral fixation	10	29 (± 5)	53 (± 7)	1.82 (± 0.14)	Mesh failure (10/10)
SERATEX^®^ E11 PA	9	71 (± 12)	199 (± 29)	3.93 (± 0.59)	Mesh failure (6/9)
					Tissue failure (3/9)
SERATEX^®^ B3 PA	10	33 (± 4)	66 (± 9)	2.11 (± 0.35)	Mesh failure (10/10)

E11 differed significantly (*p* < 0.01) regarding all evaluated variables. It achieved the highest mechanical load capacity with a maximum load of 199 ± 29 N, the greatest stiffness (3.93 ± 0.59 N/mm) and the widest displacement at failure (71 ± 12 mm).

Test groups 1 and 2 evaluated two different fixation methods of SERATEX^®^ SlimSling^®^. There were significant differences in maximum load and stiffness. With SSB, failure loads of 53 ± 7 N could be reached, which is considerably higher than loads reached with unilateral fixation (30 ± 2 N). With the double-sided fixation, stiffness was approximately twice as high as those with one-sided fixation (1.82 ± 0.14 N/mm versus 0.91 ± 0.19 N/mm). In contrast, the fixation method did not lead to a significant difference in displacement at failure (*p* > 0.05).

Comparing B3 and SSB, no significant deviation could be observed (*p* > 0.05). The same applies to the comparison between B3 and SSU concerning the displacement at failure, which achieved values of 33 ± 4 mm. Maximum load (66 ± 9 N) and stiffness (2.11 ± 0.35 N/mm) of B3 were significantly higher than in the SSU group.

**Fig. 6:**
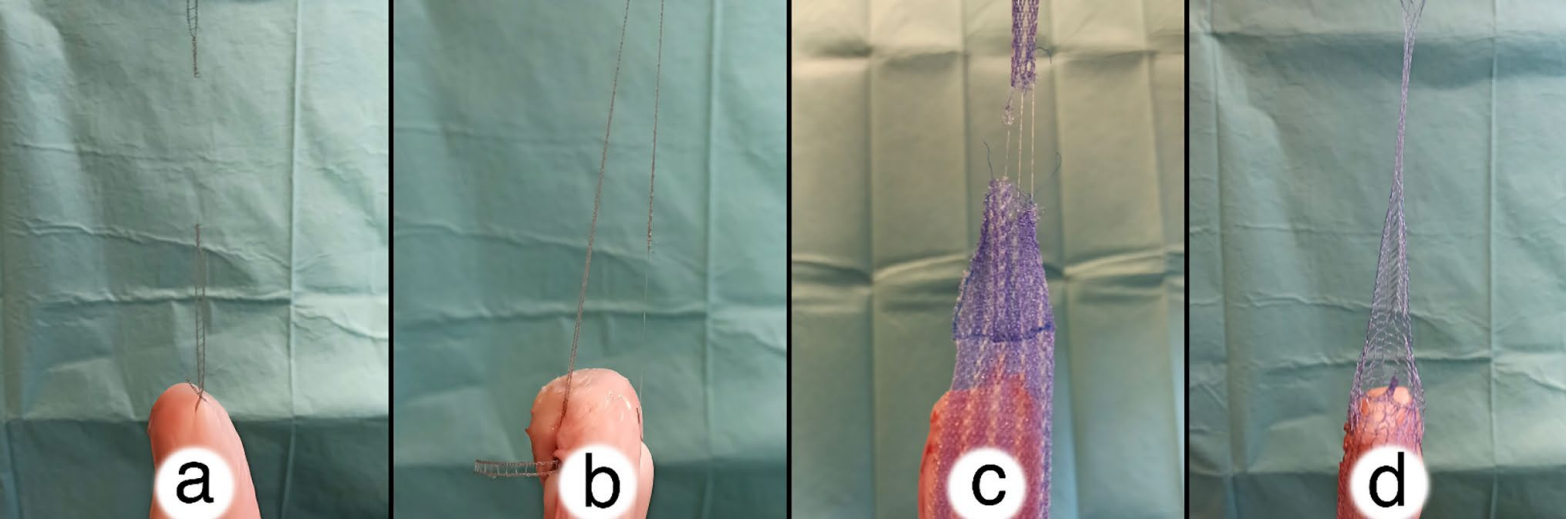
Most common failure mode (mesh failure) for all evaluated groups. **a** SERATEX^®^ SlimSling^®^ with unilateral fixation. **b** SERATEX^®^ SlimSling^®^ with bilateral fixation. **c** SERATEX^®^ E11 PA. **d** SERATEX^®^ B3 PA

**Fig. 7:**
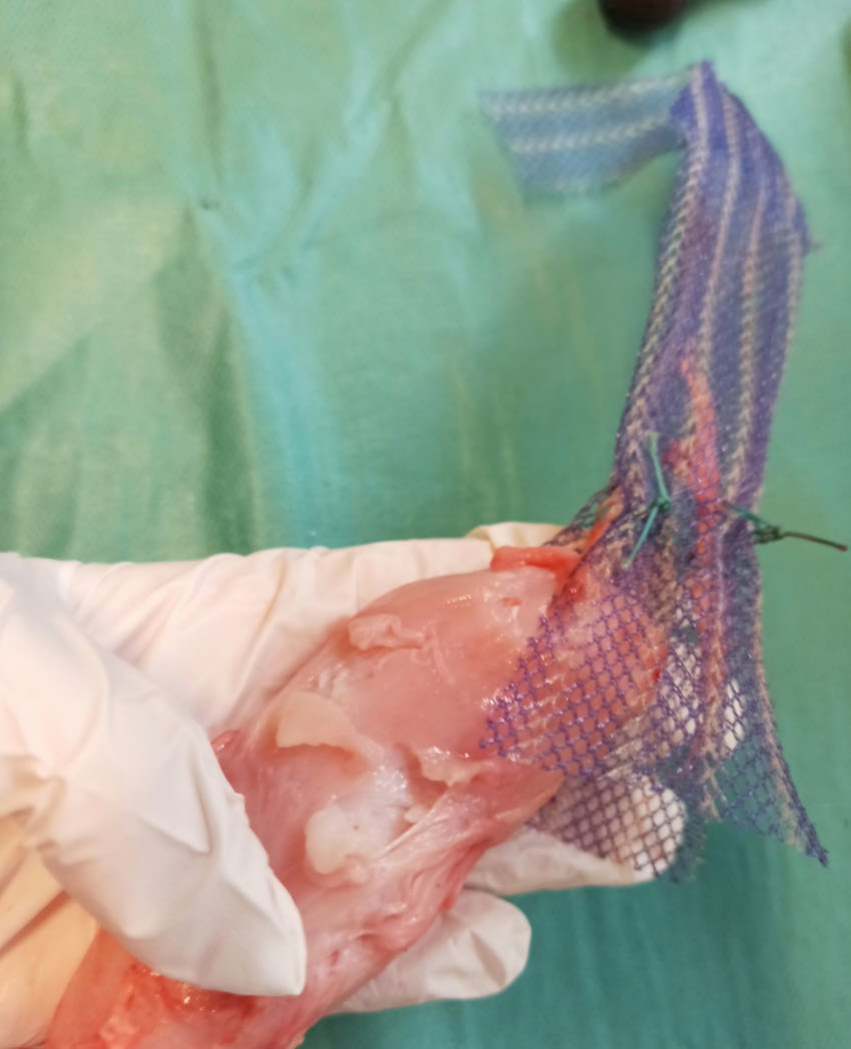
Tissue failure of SERATEX^®^ E11 PA

The mesh was the most frequent limiting factor in all four subgroups (cf. Fig. 6). Neither the vaginal tissue, nor the suture failed in all 32 trials of groups 1, 2 and 4. Only in group 3, three of the nine failures were tissue failures (cf. Fig. 7).

## Discussion

The results oppose the primary hypothesis. Meshes differ significantly regarding maximum load, displacement at failure and stiffness. The differences in all categories can be seen in Table 1.

### Maximum load

Regarding the maximum load, the mesh was the most frequent limiting factor. Thirty-eight of 41 trials showed a mesh failure. Only in three cases, a tissue failure was caused, all of them in group 3 with E11. The suture did not fail in any trial. In 2013, Zimkowski et al. described pull-out forces of 16.65 ± 3.30 N for a surgical polyester mesh (PETKM14001, Textile Development Associates) [21]. Sauerwald et al. reported failures in a similar order of magnitude, at 35 N, for SERA MESH1 PA (SERAG-WIESSNER GmbH & Co. KG, Naila, Germany) with single interrupted suture on fresh, porcine cadaver pelvises [10]. In a biomechanical study by Hachenberg et al., in vitro testing was performed on human, non-embalmed cadaver pelvises with an orthogonally placed suture at the anterior longitudinal ligament. Maximum loads of up to 80 N were achieved. Mesh attachment by Pilkinton et al. showed the highest maximum load reached with a polypropylene mesh (Boston Scientific, Boston, MA) and GORE-TEX^®^ polytetrafluoroethylene suture (W. L. Gore & Associates, Newark, DE). Maximum loads of 65.14 N, IQR 53.37–68.77 were detected [22]. Due to several differences in the trial setup, such as different test machines, variable sutures and different materials, the comparability of all studies is limited. Furthermore, the orientation of the mesh, perpendicular or parallel to the longest distance of the mesh pores, generates an altered maximum load. A perpendicular orientation leads to a greater suture retention strength [23]. Nonetheless, the recent results (30 ± 2 to 66 ± 9 N) fit in the range of data reported in literature. Only the failure loads generated by the E11 group were clearly higher, with average results of 199 ± 29 N. Larger trials should be initiated to define the required strength of the mesh. Anding et al. suggest that pull-out forces in the porcine pelvic floor of more than 50 N are not physiologically relevant [24]. According to DeLancey, the physiological force interfering on the uterus during a maximal Valsalva maneuver is also at a low level. He states that 90 g of tensile force is sufficient to provoke the same intensity of uterine descent as with an Valsalva maneuver [2]. However, in light of an unclear anatomical requirement, we should always opt for the best fixation method.

### Displacement at failure

Zimkowski et al. reported a failure of polyethylene terephthalate mesh at 21.92 ± 3.76 mm in an in vitro study [21]. Other studies described strains to failure at 36–37 mm [10]. Groups 1, 2 and 4 in this study average displacements of failure at 29–33 mm, which supports the current literature. E11 was the only mesh with a higher strain, with a mean of 71 mm. This can be explained by the textile conformation of the mesh. Conditioned by the oblique run of the fibers in relation to the tensile load, the elongation of the mesh is greater than for fibers parallel to the load.

### Stiffness

**Fig. 8:**
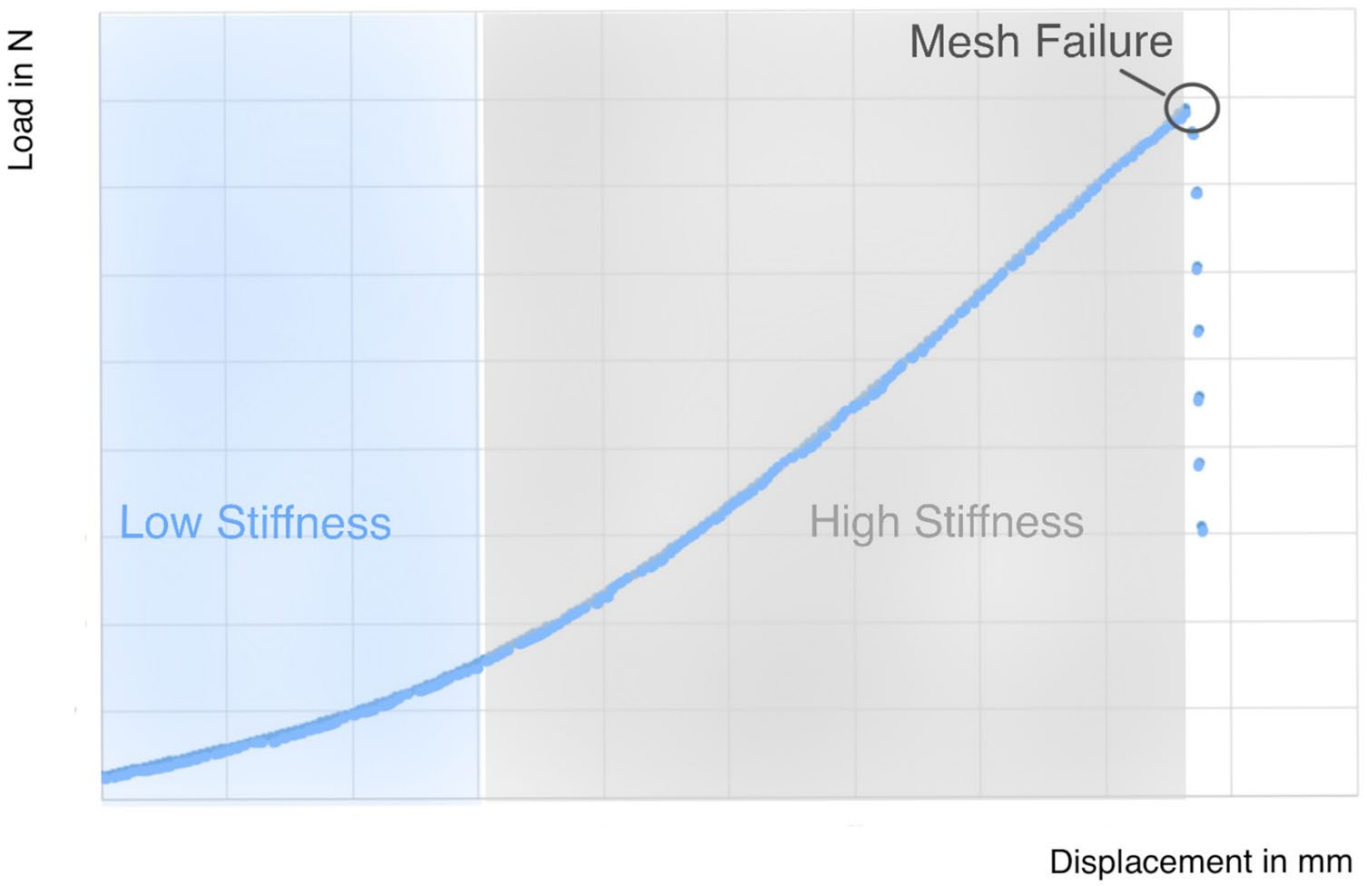
Representative force–deflection graph

The parameter stiffness, estimated by maximum load and displacement at failure, is highly contingent upon the experimental setup. It is unclear whether higher stiffness is beneficial or detrimental to surgical outcome. In the current study, a wide range of stiffness values can be observed. E11 exhibits the highest stiffness (3.93 ± 0.59 N/mm), SSU the lowest (0.91 ± 0.19 N/mm). B3 (2.11 ± 0.35 N/mm) and SSB (1.82 ± 0.14 N/mm) are in between. Advantages of greater mesh flexibility are a better adaptation to the anatomical conditions and the possibility to enable movements. However, a certain level of stiffness is necessary to keep the original shape of the mesh and to support the correct position of cervix and vagina [22]. Liang et al. compared the impact of various grades of stiffness concerning vaginal histomorphology. A higher marker for tissue injury, a thinner muscle layer and an increased collagen fraction using the stiffer (in this case 0.29 ± 0.02 N/mm, uniaxial testing) polypropylene Gynemesh^®^ PS (Ethicon, Sommersville, NJ, USA) [25] were detected. Comparability is restricted once again, as the experimental setting is not conformable. Stiffness can be calculated at different parts of the force–deflection graph, where a first period of low stiffness is followed by a higher one [26] (cf. Fig. 8). Studies which calculated the parameter at different stiffness periods cannot be directly compared from one to another. In the current study, stiffness was calculated at the higher period. Depending on the knitting pattern, the orientation of the mesh can also affect stiffness. Feola et al. implanted the mesh UltraPro (Ethicon, Somerville, NJ) in different orientations into rhesus macaques and reported a higher stiffness for an implantation parallel to the orientation lines of the mesh than for an implantation perpendicular to it [27]. Additionally, the fiber arrangement regulates the biomechanical properties of the meshes. In this study, only monofilament fibers were tested. The use of braided fibers would reduce the stiffness [28].

When selecting a mesh to repair pelvic organ prolapse, the simplicity of implementation should also be considered. SERATEX^®^ SlimSling^®^ excels in the reduced number of sutures which is especially beneficial for laparoscopic approaches and results in a shorter operating time and a lower failure rate. It can be assumed that SERATEX^®^ SlimSling^®^ goes along with the easiest laparoscopic implementation.

The use of a cadaveric model leads to some limitations. The first limitation is the exclusive use of porcine tissue. So far, comparability for human and porcine cervices have not been completely proven. In an anatomical and histological examination of the porcine vagina by Gruber et al., apart from a few differences, such as a thinner vagina with less elastin, similarity of human and porcine vagina was described [29]. Nevertheless, whilst evaluating the results, potentially different biomechanical properties of human tissue must be considered. Second, the results generated by the present experimental setup are not completely conferrable to in vivo scenarios. Since the mesh in vivo will be supported by tissue ingrowth and the effects of wound healing, this in vitro testing evaluates only the immediate post-surgical fixation instead of the long-term stability. In addition, it should be noted that the apical fixation of the mesh at the sacrum is not represented in this study. As this trial was done in uniaxial dimension only, physiologic stresses in different dimensions are not included. For a better comparability to in vivo biomechanical properties, multiaxial testing, including the apical fixation at the sacrum will be necessary for further studies. Third, a limited number of meshes and samples were analyzed. Despite the small sample size, outstanding results were achieved which would not differ considerably with a greater number of samples.

## Conclusion

This is the first biomechanical comparison between SSU, SSB, E11 and B3 used in sacrocervicopexy in a cadaver testing worldwide. Considering the data above, the mesh is the limiting factor in this biomechanical analysis despite different fixation methods. All meshes achieved a good tensile strength, but the results of the maximum load show, that E11 is superior to the other meshes and is accompanied by a higher stiffness and a longer displacement at failure. The exact biomechanical requirements for the mesh in vivo have not yet been clarified. However, an overview of the resilience of the various meshes can be very useful in everyday clinical practice, as it can enable a targeted response to the patient’s needs. Thus, if necessary, a more stable mesh can be used if higher intra-abdominal pressure is suspected. Further studies are needed to determine the actual load that the meshes must be able to withstand. SERATEX^®^ SlimSling^®^ enables a shorter operating time through a reduced number of sutures. It is a fast and simple method for the correction of pelvic organ prolapse without a loss of stability. As an effective alternative to the other tested meshes, it can facilitate the clinical routine. However, it should be fixated at two sides of the cervix to increase the maximum load.

## Data Availability

The datasets generated and analyzed during the current study are available in the OSF repository, https://osf.io/8yjf5/.

## Code availability

https://doi.org/10.17605/OSF.IO/8YJF5.
